# Whole-Genome Sequencing of Emerging Invasive Neisseria meningitidis Serogroup W in Sweden

**DOI:** 10.1128/JCM.01409-17

**Published:** 2018-03-26

**Authors:** Lorraine Eriksson, Sara Thulin Hedberg, Susanne Jacobsson, Hans Fredlund, Paula Mölling, Bianca Stenmark

**Affiliations:** a Department of Laboratory Medicine, Faculty of Medicine and Health, Örebro University, Örebro, Sweden; University Hospital Münster

**Keywords:** *Neisseria meningitidis*, serogroup W, whole-genome sequencing, invasive meningococcal disease, CC11

## Abstract

Invasive disease caused by Neisseria meningitidis serogroup W (MenW) has historically had a low incidence in Sweden, with an average incidence of 0.03 case/100,000 population from 1995 to 2014. In recent years, a significant increase in the incidence of MenW has been noted in Sweden, to an average incidence of 0.15 case/100,000 population in 2015 to 2016. In 2017 (1 January to 30 June), 33% of invasive meningococcal disease cases (7/21 cases) were caused by MenW. In the present study, all invasive MenW isolates from Sweden collected in 1995 to June 2017 (*n* = 86) were subjected to whole-genome sequencing to determine the population structure and to compare isolates from Sweden with historical and international cases. The increase of MenW in Sweden was determined to be due to isolates belonging to the South American sublineage of MenW clonal complex 11, namely, the novel U.K. 2013 lineage. This lineage was introduced in Sweden in 2013 and has since been the dominant lineage of MenW.

## INTRODUCTION

Neisseria meningitidis is a human commensal bacterium of the upper respiratory tract, as well as a pathogen that can cause meningitis and septicemia. The rate of death due to invasive meningococcal disease (IMD) is approximately 8%, even though adequate antibiotics and vaccines are available (1–3). N. meningitidis is divided into 12 serogroups, depending on expression of the polysaccharide capsule, and the majority of IMD is caused by serogroups A, B, C, W, X, and Y, with differences in serogroup distributions in different countries (4). Based on multilocus sequence typing (MLST), genetically similar meningococci may be further clustered into clonal complexes (CCs), some of which (for example, CC11, CC32, and CC41/44) are known to contain more invasive or hypervirulent isolates (4–6).

The first global epidemic caused by N. meningitidis serogroup W (MenW) was in 2000, after the Hajj pilgrimage to Mecca, Saudi Arabia, when IMD was reported among pilgrims and close contacts from several countries (7). The disease was attributed to a specific MenW lineage belonging to CC11, referred to as the Hajj lineage (8), and subsequent dissemination of this lineage was reported in several countries, including Sweden (7, 9). Since the Hajj outbreak, MenW has been associated with outbreaks in several African (10–12) and South American (13) countries. In recent years, some countries have reported an increased incidence of MenW (5, 14–17). Disease-causing MenW CC11 belongs to the CC11 lineage 11.1, which can be divided into the Hajj and South American sublineages (5). The South American sublineage emerged in the mid-2000s with the spread of the South American lineage in Brazil, Argentina, and Chile (13). The South American sublineage has been further divided into the original U.K. lineage, which emerged in 2009 (5, 13), and the newly emerging novel U.K. 2013 lineage (18). IMD caused by MenW has been increasing in England and Wales since 2009, and this increase has been mainly due to the novel U.K. 2013 lineage since its emergence in 2013 (18). In 2015, the novel U.K. 2013 lineage was involved in an outbreak of MenW at the World Scout Jamboree in Japan, with 2 confirmed invasive cases from Sweden and 4 cases from Scotland (18, 19).

Historically, serogroups B and C have caused most cases of IMD in Sweden; in recent years, however, there has been a shift in incidence to serogroups that previously rarely caused IMD. There was an increase in serogroup Y in 2006 (20, 21), followed by an increase in MenW since 2015. Before their emergence, these serogroups were linked to lower prevalence and were represented by only a few cases of IMD in Sweden each year (22, 23). The aim of this study was to characterize epidemiologically and genetically and to compare all invasive MenW isolates in Sweden from 1995 to June 2017, in order to determine the population structure, in comparison with historical and international cases, as part of understanding the basis of the increased MenW incidence in Europe.

## MATERIALS AND METHODS

### Bacterial isolates and DNA extraction.

In Sweden, all invasive cases of meningococcal disease, according to the European Union case definition, are mandatorily reported by clinicians to the Public Health Agency of Sweden (24) and the corresponding isolates are sent to the National Reference Laboratory for Neisseria meningitidis at Örebro University Hospital. The bacteria are subsequently cultured overnight on chocolate agar plates at 37°C in 5% CO_2_, serogrouped by coagglutination (25), and stored at −70°C. In this study, all invasive MenW isolates collected in Sweden between 1 January 1995 and 30 June 2017 (*n* = 86) were subjected to whole-genome sequencing (WGS). DNA from cultured isolates was automatically extracted with the QIAsymphony system (Qiagen, Hilden, Germany) using the QIAsymphony DSP virus/pathogen kit, according to the manufacturer's instructions, with added RNase treatment and elution with Tris-HCl (pH 8).

### Library preparation and sequencing.

Sequencing libraries were constructed using the Nextera XT DNA library preparation kit (Illumina, San Diego, CA), with slight modifications of the protocol in order to optimize the average fragment length. The tagmentation was performed with a lower DNA input (0.75 ng), and the tagmentation time was increased to 7.5 min. Amplification of the tagmented DNA was performed using index primers, and the amplified products were purified manually or automatically with the ACSIA NGS^LibPrep^ edition (PrimaDiag, Romainville, France), using AMPure XP beads (Beckman Coulter, Brea, CA). The normalization and pooling were performed manually or automatically using the ACSIA liquid-handling robot, based on the size of each fragment determined with a TapeStation 4200 system (Agilent, Santa Clara, CA), and the DNA concentration was measured with Qubit (Thermo Fisher, Waltham, MA). Sequencing was performed with an Illumina MiSeq instrument and a MiSeq reagent kit v3, 600 cycles (Illumina), according to the manufacturer's instructions. The reads were assembled *de novo* using Velvet (26). The quality of sequencing was controlled by the *N*_50_ value, contig count, and coverage. The sequences were trimmed until the average base quality (Phred score) was >30 in a window of 20 bases. The assemblies were uploaded to the Neisseria PubMLST database, and the sequences were automatically scanned and tagged against defined “NEIS” loci in the database (27). The corresponding identification numbers and additional epidemiological information on the isolates from Sweden are presented in Table S1 in the supplemental material.

### Genome comparisons.

All 86 invasive MenW isolates were sequenced with 55- to 163-fold coverage, *N*_50_ values of 19,024 to 59,410, and contig counts of 136 to 407 and were deposited in the Neisseria PubMLST database. Genomes used for the comparisons were publicly available in the Neisseria PubMLST database (http://PubMLST.org/neisseria). Genome comparisons were performed using the PubMLST genome comparator tool. The 1,605 N. meningitidis core loci (present in ≥95% of the N. meningitidis isolates in the Neisseria PubMLST database) in N. meningitidis cgMLST v1.0 (27) were used for all comparisons between genomes. The genome comparator tool automatically generates distance matrices based on the number of variable alleles in the core loci, which can be visualized in a neighbor-net network with SplitsTree4 (http://www.splitstree.org) (28). Isolates from Sweden were compared to international MenW CC11 isolates present in the PubMLST database (*n* = 1,088, as of 25 May 2017). In some comparisons, 11 carrier isolates from Sweden that had been sequenced and deposited in PubMLST during a previous study were included (18). To further distinguish the genomes of isolates from Sweden within the different clusters, genomes with similar core genome MLST (cgMLST) profiles were searched using the predefined classification schemes in the Neisseria PubMLST database. Incomplete loci (due to incomplete assembly) were ignored in pairwise comparisons in the distance matrix calculations. Isolates included in the classification schemes were grouped based on their core genome sequence type (cgST). In order to be assigned to a cgST, isolates needed to have >1,555 complete cgMLST loci, which led to the exclusion of all isolates with sequence quality below this threshold. The classification schemes Nm_cgc_100 (accessed 12 July 2017) and Nm_cgc_25 (accessed 12 July 2017), representing all genomes with <100 and <25 allelic differences, respectively, compared to the chosen genome (identification no. 45025), were used.

### Statistical analyses.

A two-tailed Mann-Whitney U test was used to calculate differences in age between different lineages. Statistically significant trends in incidence were tested using the Joinpoint v4.5.0.1 regression program (29). The annual percentage change in MenW incidence was calculated with a logarithmic linear regression model that added a minimal number of joinpoints and calculated the difference up to statistically significant levels, using the Monte Carlo permutation test (30) (Fig. S1). A significant increase in incidence was defined as the slope of the curve being statistically significant (*P* < 0.05).

## RESULTS

Eighty-three invasive MenW isolates were included in the present study. Three of the isolates serogrouped as MenW, with fine type P1.5-2, 10-1, F4-1, sequence type 23 (ST-23) (CC23), were confirmed to contain a mutation in the amino acid at position 310 of the capsule polymerase enzyme *csy* that led to a change of glycine (serogroup Y) or proline (serogroup W) to serine, enabling expression of a mixed galactose/glucose-sialic acid polysaccharide. Because these isolates thus belong to serogroup W/Y (31–33), they were excluded from further analyses.

**FIG 1:**
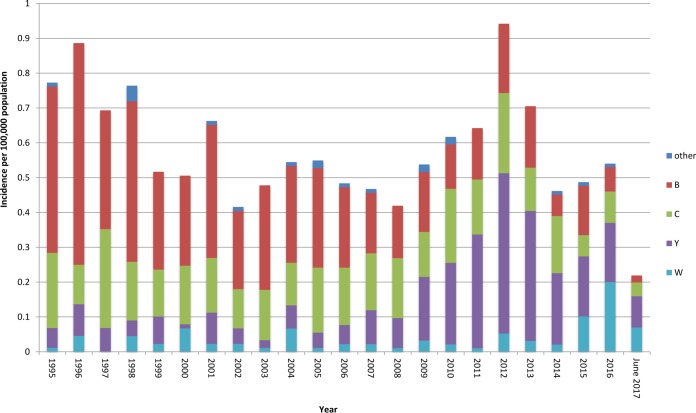
Incidence of invasive Neisseria meningitidis disease in Sweden, by serogroup, from 1995 to June 2017.

**FIG 2:**
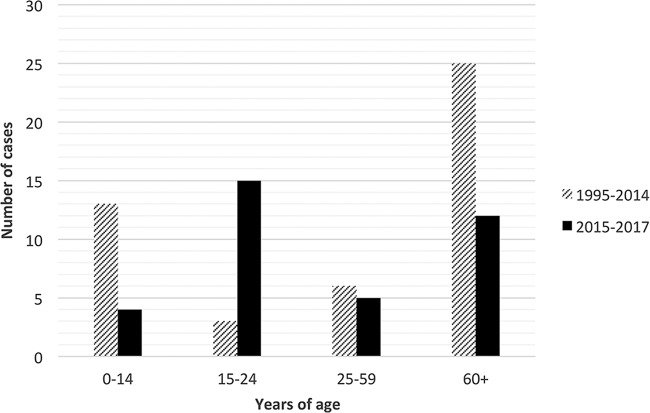
Numbers of serogroup W IMD cases in different age groups for the periods 1995 to 2014 and 2015 to 2017.

The incidence of the four most prevalent serogroups in Sweden between 1995 to 2017 is shown in Fig. 1. There was a statistically significant increase (*P* < 0.05) in the incidence of MenW between the period of 1995 to 2014 (average incidence of 0.03 case/100,000 population) and the period of 2015 to 2016 (average incidence of 0.15 case/100,000 population) (see Fig. S1 in the supplemental material). In June 2017, MenW represented 33% of all IMD reported. The median age of patients with MenW IMD decreased after the increased incidence (Fig. 2), from 60 years in 1995 to 2014 (interquartile range [IQR], 3.5 to 76 years) to 23 years in 2015 to June 2017 (IQR, 18 to 72 years); however, this decrease was not significant (*P* = 0.79). Since 2010, there have been 6 fatal cases of MenW disease in total, 3 in 2015 and 3 in 2016.

**FIG 3:**
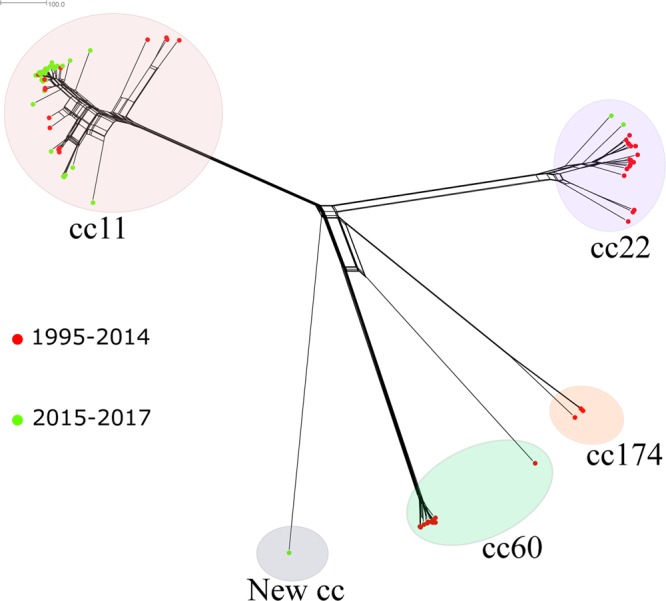
Neighbor-net network based on 1,605 core genes, displaying invasive serogroup W isolates from Sweden collected in 1995 to June 2017 (*n* = 83). Circles represent the different clonal complexes, and isolates are indicted by colored dots. Isolates depicted with red dots were collected in 1995 to 2014 (*n* = 46), and isolates depicted with green dots were collected in 2015 to 2017 (*n* = 37). The distance between isolates was defined as the number of loci with allelic differences.

The population structure based on the N. meningitidis core loci (*n* = 1,605) of MenW genomes in Sweden (*n* = 83) from 1995 to June 2017 is shown in Fig. 3. The majority of MenW isolates from Sweden belonged to CC11 (59% [*n* = 49]), followed by CC22 (23% [*n* = 19]), CC60 (13% [*n* = 11]), and CC174 (4% [*n* = 3]), with 1 isolate of ST-12019 not assigned to any CC. An increase in the proportion of MenW CC11 was noted; CC11 represented 8/10 (80%) of all MenW cases in 2015, 19/20 cases (95%) in 2016, and 7/7 cases (100%) in 2017 (January to June).

**FIG 4:**
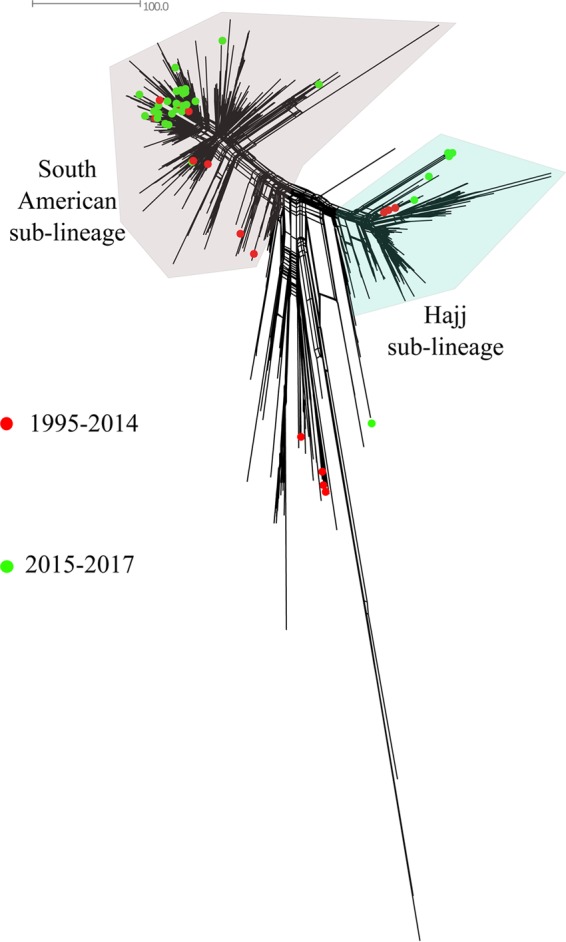
Neighbor-net network based on 1,605 core genes for serogroup W CC11 isolates in the Neisseria PubMLST database. Red dots represent invasive isolates from Sweden collected in 1995 to 2014 (*n* = 15), and green dots represent isolates collected in 2015 to 2017 (*n* = 34). The distance between isolates was defined as the number of loci with allelic differences.

Since 2015, CC11 has been the most prevalent CC for MenW (Fig. 3). When the MenW CC11 isolates from Sweden (*n* = 49) were compared to all MenW CC11 isolates in the Neisseria PubMLST database, including isolates from 12 African countries (*n* = 241), 11 European countries (*n* = 807), 2 Asian countries (*n* = 5), 2 South American countries (*n* = 11), and 2 North American countries (*n* = 24) (Fig. 4), the majority of invasive isolates from Sweden (35/49 isolates [71%]) clustered with the South American sublineage (collected in 2005, 2008, and 2011 to 2017). Nine isolates from Sweden (collected in 2000, 2002, and 2015 to 2017) clustered with the Hajj sublineage, which contains isolates associated with outbreaks in Burkina Faso (2001, 2002, and 2004), South Africa (2003 to 2013), and northern Africa (2000 to 2003). Three of the isolates from Sweden in this sublineage were collected in 2017, and the cases were epidemiologically linked. The differences between the Hajj and South American sublineages were approximately 99 loci with allelic differences among the 1,605 core genes used for comparative genomic analysis. While most MenW CC11 isolates from Sweden clustered within the Hajj sublineage or the South American sublineage, 5 isolates did not cluster in either (Fig. 4). Those isolates were the first isolates collected in Sweden that belonged to CC11, collected in 1998 (*n* = 3), 2010 (*n* = 1), and 2016 (*n* = 1).

**FIG 5:**
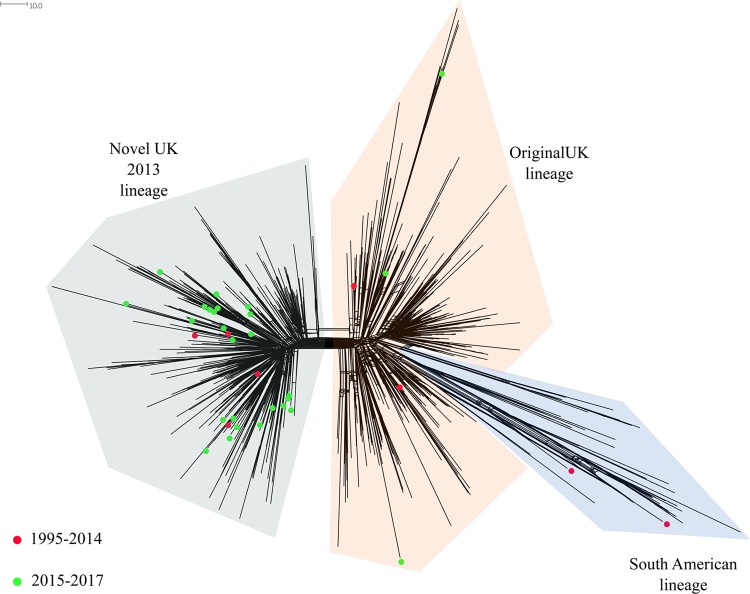
Neighbor-net network based on 1,605 core genes for isolates belonging to the South American lineage, the original U.K. lineage, and the novel U.K. 2013 lineage, which all belong to the South American sublineage of serogroup W CC11. MenW CC11 invasive isolates from Sweden collected in 1995 to 2014 (*n* = 8) are shown as red dots, and isolates collected in 2015 to 2017 (*n* = 27) are shown as green dots. The distance between isolates was defined as the number of loci with allelic differences.

In order to study the lineages of the South American sublineage, namely, the original U.K. lineage and the novel U.K. 2013 lineage, a neighbor-net network was created of 714 isolates from the Neisseria PubMLST database (of which 35 were from Sweden) that had ≤100 allelic differences, compared to a randomly chosen isolate from Sweden (Nm_cgc_100 group 165) belonging to the South American sublineage (Fig. 5). The isolates from the PubMLST database were from 5 African countries (*n* = 13), 10 European countries (*n* = 641), 1 South American country (*n* = 2), and 2 North American countries (*n* = 23). Only 2 isolates from Sweden (collected in 2005 and 2008) clustered with the South American lineage, and 5 isolates from Sweden (collected in 2011, 2012, and 2016) clustered with the original U.K. lineage. When the lineages within the South American sublineage were compared, approximately 60 loci with allelic differences, of the 1,605 core loci, separated the original U.K. lineage from the South American lineage (Fig. 5). The remaining isolates from Sweden (*n* = 28), all collected in 2013 to 2016, clustered with the novel U.K. 2013 lineage.

**FIG 6:**
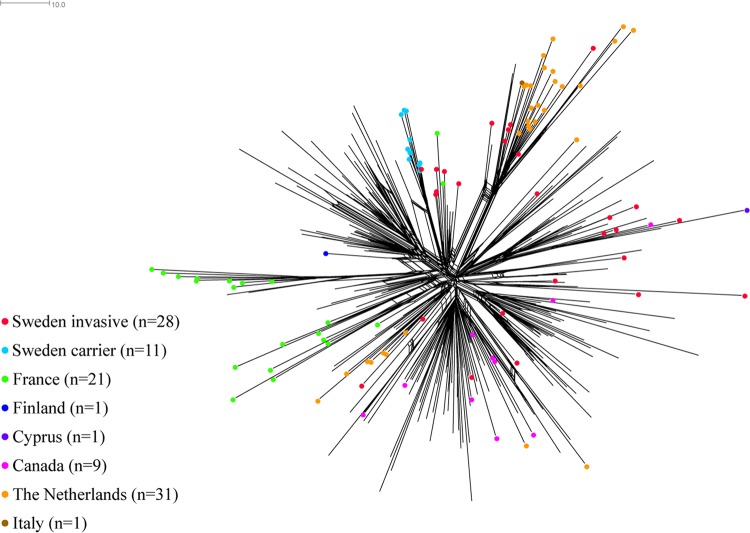
Neighbor-net network based on 1,605 core genes for isolates representing the novel U.K. 2013 lineage of MenW CC11. Invasive isolates from Sweden (*n* = 28) are shown as red dots, and carrier isolates from Sweden (*n* = 11) are shown as light blue dots. U.K. isolates (*n* = 211) are unmarked. The distance between isolates was defined as the number of loci with allelic differences.

The isolates related to the novel U.K. 2013 lineage were compared by using isolates deposited in the Neisseria PubMLST database with ≤25 allelic mismatches, compared with a randomly chosen isolate from Sweden belonging to the novel U.K. 2013 lineage (*n* = 314) (Nm_cgc_25 group 1376). The neighbor-net network spanned the novel U.K. 2013 lineage and included 28 of the invasive isolates from Sweden (collected in 2013 to 2017) sequenced in the present study (Fig. 6), as well as 11 carrier isolates from Sweden that were sequenced and deposited in PubMLST during a previous study on the World Scout Jamboree in Japan in 2015 (18). Six invasive isolates from Sweden, collected in 2015 to 2017, clustered on the same branch as 22 isolates from the Netherlands, 3 from the United Kingdom, and 1 from Italy, all of which were collected in 2015 and 2016. No country-specific clusters were detected. Among the isolates from Sweden, the same allelic differences in 21 genes that had been described previously by Lucidarme et al. (18) were detected between the novel U.K. 2013 lineage and the original U.K. lineage.

**FIG 7:**
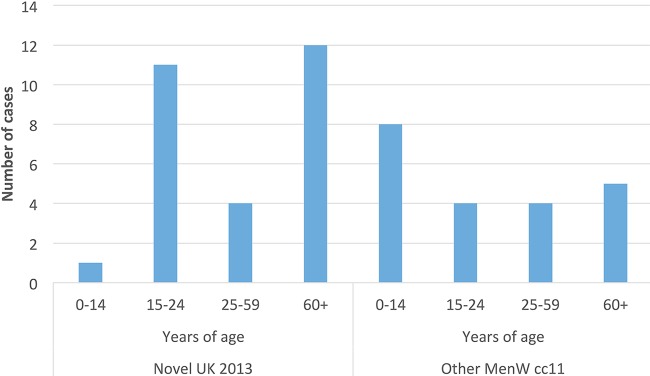
Number of IMD cases caused by the novel U.K. 2013 lineage and all other MenW CC11 strains in 1995 to June 2017.

**FIG 8:**
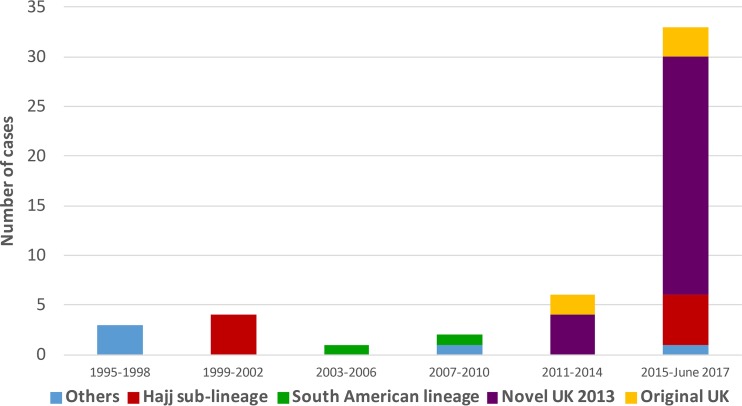
Distribution of lineages among serogroup W isolates in Sweden (*n* = 83) from 1995 to June 2017.

The age distribution was significantly higher (*P* = 0.04) for patients with IMD caused by the novel U.K. 2013 lineage (median age, 51.5 years [IQR, 18.5 to 72 years]) than for patients with IMD caused by all other CC11 isolates (median age, 22 years [IQR, 3.5 to 54 years]). However, an increase among individuals 15 to 24 years of age was also noted in the novel U.K. 2013 lineage (Fig. 7). In addition, 6 fatal cases of MenW disease since 2010 occurred in 2015 and 2016 (patient ages, 18, 23, 51, 57, 84, and 90 years). Five of the 6 deaths were caused by isolates belonging to the novel U.K. 2013 lineage, which dominated during 2015 and 2016 (Fig. 8).

## DISCUSSION

A significant increase in MenW disease has been observed in Sweden; the incidence has increased 10-fold from 2014 to 2016 (from 0.02 to 0.2 cases per 100,000 population). The present study on all invasive MenW isolates in Sweden between 1995 and June 2017 suggests that the increase of MenW in Sweden is due to the increase of a lineage within the South American sublineage, referred to as the novel U.K. 2013 lineage. During the years investigated, the original U.K. lineage caused only a few sporadic cases, suggesting a less successful spread in Sweden, compared to the novel U.K. 2013 lineage. Also, the Hajj sublineage has been uncommon in Sweden, with only 9 cases reported during the 23-year period investigated in this study. Although it is predominant among U.K. isolates, the range of countries with isolates belonging to the novel U.K. 2013 lineage in the Neisseria PubMLST database suggests that this strain is prevalent in a wide geographical area (Fig. 6).

There was a tendency for a shift into the age group of 15 to 24 years after the MenW increase in Sweden (Fig. 2). Although it is difficult to make assessments about the age group distribution of MenW between the different lineages, due to the small number of cases, there was a significant increase of the novel U.K. 2013 lineage in the ≥60-year age group, as well as a peak among younger adults (Fig. 7), in accordance with previous studies (34, 35). The median age of patients with IMD caused by serogroup Y (collected in 1995 to 2012) was 62 years (36), suggesting that the lineages causing increases of both MenW and serogroup Y are causing disease in an older age group.

The reasons for the increase of MenW in Sweden and other countries are still unclear, but a recent carriage study from the United Kingdom that was performed at the University of Nottingham in 2015 and 2016 (37) showed a large increase of MenW carriage in adolescents, from 14% in 2015 to 46% in 2016. In that carriage study, it was discovered that the carriage isolates were similar (identical PorA and factor H binding protein) to the invasive MenW isolates that are currently spreading in England and Wales (37). A carriage study on the overall population has not been performed in Sweden, and the MenW carriage state in Sweden remains undetermined. In this study, a small group of carrier isolates from Sweden, previously reported in a study by Lucidarme et al. (18) of an outbreak at a World Scout Jamboree in Japan 2015, were included. A carriage study on the overall population in Sweden would be of importance, since an increase in carriage of MenW could lead to spread within and between countries and also to increased risk of invasive disease. In order to halt this increase of MenW in the United Kingdom, the quadrivalent ACWY conjugate vaccine has recently been introduced to adolescents and new university entrants (35). Another concern with the emergence of MenW is the atypical symptoms that have been reported, including gastrointestinal symptoms, septic arthritis, necrotizing fasciitis, pneumonia, and pericarditis (14, 38, 39), which could lead to incorrect clinical diagnoses. The results from the present study suggest that the same lineage has increased in Sweden and in the United Kingdom, which suggests that the problems with atypical symptoms and incorrect diagnoses might have arisen in Sweden as well. The clinical presentation of patients with IMD is not mandatorily reported to the Swedish Reference Laboratory; therefore, this association could not be determined for the MenW cases from Sweden. However, the disease manifestations of invasive disease caused by serogroup Y in Sweden were investigated in a previous study (36), and a similar study on MenW would be of interest in order to determine whether disease caused by this serogroup is associated with similar atypical symptoms in Sweden.

Due to the low incidence of IMD in Sweden since the 1990s (<1 case/100,000 population), the number of MenW cases included in this study is small and thus the diversity analysis of the older isolates is based on a limited number of isolates. Also, the comparisons of genomes within different cgMLST classification schemes were restricted to genomes with high sequencing quality, which might have led to genomes being excluded from the analyses. In addition, there are presently only a few publicly available MenW genomes representing the recent increase of MenW in Europe, and the genomes deposited in the Neisseria PubMLST database are overrepresented by European countries, with routine typing based on WGS. Therefore, the isolates from Sweden and the prevalence of the novel U.K. 2013 lineage in surrounding countries could not be completely established in the present study.

In conclusion, this study indicated that the recent increase of MenW disease in Sweden was due to the novel U.K. 2013 lineage, belonging to the CC11 South American sublineage. Continued monitoring of the incidence of MenW in Sweden is warranted, especially if atypical presentations of IMD would delay the diagnosis. If the incidence continues to increase, then the introduction of general vaccination against MenW in Sweden could be considered. To further understand the increase in MenW IMD in Sweden, a carriage study, possibly giving information of the transmission of MenW in the population, would be valuable.

## Supplementary Material

Supplemental material
