# Supplementary material for: Whole-Genome Sequencing of Emerging Invasive Neisseria meningitidis Serogroup W in Sweden
Source: J Clin Microbiol. 2018 Mar 26;56(4):e01409-17. doi: 10.1128/JCM.01409-17 (PMC5869829; doi:10.1128/JCM.01409-17)
Supplement: Supplemental material [file supp_56_4_e01409-17__index.html]

Whole-Genome Sequencing of Emerging Invasive Neisseria meningitidis Serogroup W in Sweden — Supplemental material 

# Whole-Genome Sequencing of Emerging Invasive Neisseria meningitidis Serogroup W in Sweden

## Supplemental material

- Supplemental file 1 -

  Fig. S1 (Trends in MenW incidence from 1994 to 2016 as determined using joinpoint regression analysis)

  PDF, 112K
- Supplemental file 2 -

  Table S1 (Molecular characterization and epidemiological data for the invasive WGS serogroup W isolates from Sweden)

  PDF, 160K
