# Supplementary material for: Whole-Genome Sequencing of Emerging Invasive Neisseria meningitidis Serogroup W in Sweden
Source: J Clin Microbiol. 2018 Mar 26;56(4):e01409-17. doi: 10.1128/JCM.01409-17 (PMC5869829; doi:10.1128/JCM.01409-17)
Supplement: Supplemental material [file JCM.01409-17_zjm999095870s1.pdf]

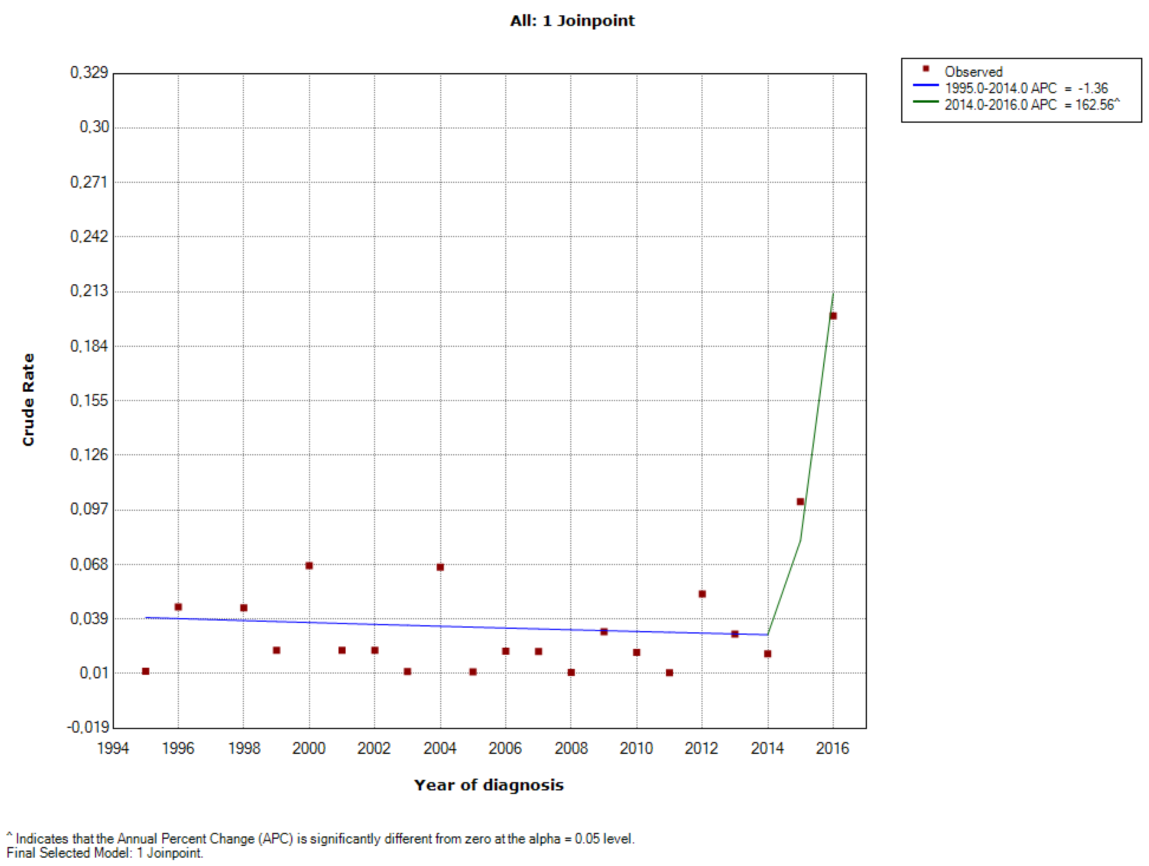

Fig S1. Trends in MenW incidence from 1994 to 2016 (no cases in 1997) using joinpoint regression analysis. ^ indicates that the annual percent change (APC) is significantly different.
