# Supplementary material for: Whole-Genome Sequencing of Emerging Invasive Neisseria meningitidis Serogroup W in Sweden
Source: J Clin Microbiol. 2018 Mar 26;56(4):e01409-17. doi: 10.1128/JCM.01409-17 (PMC5869829; doi:10.1128/JCM.01409-17)
Supplement: Supplemental material [file JCM.01409-17_zjm999095870s2.pdf]

Table S1. Molecular characterization and epidemiological data for the invasive WGS serogroup W isolates from Sweden (n=83).

PubMLST ID	Finetype	Clonal complex (cc)	Serogroup	Lineage	Year
26067	P1.18-1,3; F4-1; ST-22	cc22	W		1995
38602	P1.5,2; F1-1; ST-11	cc11	W	Novel UK 2013 lineage	2015
38614	P1.5-2; F1-1; ST-11	cc11	W	Novel UK 2013 lineage	2015
38615	P1.5-2; F1-1; ST-11	cc11	W	Novel UK 2013 lineage	2015
38690	P1.5-2; F1-1; ST-11	cc11	W	Novel UK 2013 lineage	2015
38691	P1.5-2; F1-1; ST-11	cc11	W	Novel UK 2013 lineage	2015
38692	P1.5-2; F1-1; ST-11	cc11	W	Novel UK 2013 lineage	2015
38693	P1.17-9; F1-7; ST-12019	New	W		2015
38694	P1.5-2; F1-1; ST-11	cc11	W	Novel UK 2013 lineage	2015
39601	P1.5-2; F1-7; ST-11	cc11	W	Original UK lineage	2017
39602	P1.5-2; F1-1; ST-11	cc11	W	Novel UK 2013 lineage	2017
39603	P1.5-2; F1-1; ST-11	cc11	W	Novel UK 2013 lineage	2017
41963	P1.5-2; F1-1; ST-11	cc11	W	Novel UK 2013 lineage	2016
41964	P1.5-2; F1-1; ST-11	cc11	W	Novel UK 2013 lineage	2016
41966	P1.5-2; F1-1; ST-11	cc11	W	Original UK	2016
42320	P1.18-1,3; F3-9; ST-22	cc22	W		2013
42336	P1.18-1,3; F3-9; ST-22	cc22	W		2013
42363	P1.5-2; F1-1; ST-11	cc11	W	Novel UK 2013 lineage	2014
42391	P1.5-2; F1-1; ST-11	cc11	W	Novel UK 2013 lineage	2014
42420	P1.5-2; F1-1; ST-11	cc11	W	Novel UK 2013 lineage	2016
42423	P1.18-1,3; F4-1; ST-22	cc22	W		2015
42424	P1.5-2; F1-1; ST-11	cc11	W	Novel UK 2013 lineage	2016
42425	P1.5-2; F1-1; ST-11	cc11	W	Hajj	2015
42436	P1.5-2; F1-1; ST-11	cc11	W	Novel UK 2013 lineage	2016
42445	P1.5-2; F1-1; ST-11	cc11	W	Novel UK 2013 lineage	2016
42786	P1.5-2; F1-1; ST-11	cc11	W	Novel UK 2013 lineage	2016
45025	P1.5-2; F1-1; ST-11	cc11	W	Novel UK 2013 lineage	2016
46377	P1.5-2; F1-1; ST-11	cc11	W	Novel UK 2013 lineage	2016
46378	P1.5-2; F1-1; ST-11	cc11	W	Novel UK 2013 lineage	2016
47166	P1.5-2; F1-1; ST-11	cc11	W	Novel UK 2013 lineage	2016
47167	P1.5-2; F1-1; ST-11	cc11	W	Novel UK 2013 lineage	2016
47170	P1.18-1,3; F4-1; ST-22	cc22	W		2016
47172	P1.5-1,2-2; F3-9; ST-174	cc174	W		1996
47173	P1.5-1,2-2; F3-9; ST-174	cc174	W		1996
47174	P1.18-1,3; F3-9, ST-174	cc174	W		1996
47175	P1.5-2; F1-1; ST-11	cc11	W	Other	1998
47176	P1.18-1,3; F4-1; ST-22	cc22	W		1998
47177	P1.5-2; F4-1; ST-11	cc11	W	Other	1998
47178	P1.5-2;F1-14; ST-11	cc11	W	Other	1998
47179	P1.5-2; F5-7; ST-60	cc60	W		2000
47180	P1.5-2, F1-1; ST-11	cc11	W	Hajj sub-lineage	2000
47181	P1.5-2; F1-1; ST-11	cc11	W	Hajj sub-lineage	2000
47182	P1.5-2; F1-1; ST-11	cc11	W	Hajj sub-lineage	2000
47184	P1.18-1,3; F4-1; ST-22	cc22	W		2001

47185	P1.5-1,2-5; F5-8; ST-22	cc22	W		2001
47186	P1.5-2; F1-1; ST-11	cc11	W	Hajj sub-lineage	2002
47187	P1.5-2; F5-7; ST-60	cc60	W		2002
47188	P1.5-2; F5-7; ST-60	cc60	W		2003
47189	P1.5-2; F5-7; ST-60	cc60	W		2004
47190	P1.18-1,3; F4-1; ST-22	cc22	W		2004
47191	P1.18-1,3; F1-5; ST-22	cc22	W		2004
47192	P1.5-2; F5-7; ST-60	cc60	W		2004
47193	P1.18-1,3; F4-1; ST-22	cc22	W		2004
47194	P1.5-2; F5-7; ST-60	cc60	W		2004
47195	P1.5-2; F1-68; ST-11	cc11	W	South American sub-lineage	2005
47196	P1.5-2; F5-7; ST-60	cc60	W		2006
47197	P1.18-1,3; F5-5; ST-22	cc22	W		2006
47198	P1.18-1,3; F5-5; ST-22	cc22	W		2007
47199	P1.5-2; F5-7; ST-60	cc60	W		2007
47200	P1.5-2; F1-1; ST-11	cc11	W	South American sub-lineage	2008
47201	P1.18-1,3; F3-9; ST-22	cc22	W		2009
47202	P1.21-26; F5-2; ST-60	cc60	W		2009
47204	P1.5-2; F1-14; ST-11	cc11	W	Other	2010
47205	P1.18-1,3; F3-9; ST-22	cc22	W		2010
47206	P1.5-2; F1-1; ST-11	cc11	W	Original UK lineage	2011
47207	P1.18-1,3; F3-9; ST-22	cc22	W		2012
47208	P1.5-2; F1-1; ST-11	cc11	W	Original UK lineage	2012
47209	P1.5-2; F5-7; ST-60	cc60	W		2012
47210	P1.18-1,3; F1-6; ST-22	cc22	W		2012
47211	P1.5-2; F5-7; ST-60	cc60	W		2012
50863	P1.5-2; F1-1; ST-11	cc11	W	Novel UK 2013 lineage	2016
50865	P1.5-2; F1-1; ST-11	cc11	W	Novel UK 2013 lineage	2016
50872	P1.5-2; F1-1; ST-11	cc11	W	Original UK lineage	2016
50875	P1.5-2; F1-1; ST-11	cc11	W	Other	2016
50975	P1.5-2; F5-173; ST-11	cc11	W	Hajj sub-lineage	2016
51910	P1.5-2; F1-1; ST-11	cc11	W	Novel UK 2013 lineage	2017
52796	P1.5-2; F1-1; ST-11	cc11	W	Hajj sub-lineage	2017
52797	P1.5-2; F1-1; ST-11	cc11	W	Hajj sub-lineage	2017
53033	P1.5-2; F1-1; ST-11	cc11	W	Novel UK 2013 lineage	2013
53042	P1.7-1,4-1; F4-1; ST-22	cc22	W		1999
53043	P1.18-1,3; F4-1; ST-22	cc22	W		1999
53776	P1.5-2; F1-1; ST-11	cc11	W	Hajj sub-lineage	2017
50876	P1.5-2; F1-1; ST-11	cc11	W	Novel UK 2013 lineage	2016
